# Reimagining Cannabidiol Delivery Through Carbon Nanodots

**DOI:** 10.3390/molecules31193404

**Published:** 2026-09-24

**Authors:** Iddrisu Ibrahim, Kelci Lawrence, Othreniel Angel Forte, Navitri Chandra Naidu, Latrell Huitt, Nathaniel Ajibola, Junhuan Xu, Robertson K. Boakai, Moses Mengu, Fortune Akabanda, James Owusu-Kwarteng, Olufemi S. Ajayi, Joseph Atia Ayariga

**Affiliations:** 1 The Microbiology Program, College of Science, Technology, Engineering, and Mathematics (C-STEM), Alabama State University, Montgomery, AL 36104, USA; iiddrisu3715@alasu.edu (I.I.); klawrence0670@alasu.edu (K.L.); oforte3773@myasu.alasu.edu (O.A.F.); nnaidu@alasu.edu (N.C.N.); lhuitt@alasu.edu (L.H.); najibola7327@alasu.edu (N.A.); jxu@alasu.edu (J.X.); brobertson@alasu.edu (R.K.B.); 2 The Industrial Hemp Program, College of Science, Technology, Engineering, and Mathematics (C-STEM), Alabama State University, Montgomery, AL 36104, USA; 3 African Drug Discovery and Innovation Network, Sunyani P.O. Box AB45, Ghana; mosesmengu@gmail.com (M.M.); james.owusu-kwarteng@uenr.edu.gh (J.O.-K.); 4 Council for Scientific and Industrial Research (CSIR), College of Science and Technology (CCST), Josip Broz Tito Avenue (GL-063-0751), Cantonments, Accra P.O. Box M 32, Ghana; 5 Department of Food Science and Technology, University for Development Studies, Tamale P.O. Box TL1350, Ghana; fakabanda@uds.edu.gh; 6 Department of Food Science and Technology, School of Agriculture and Technology, University of Energy and Natural Resources, Sunyani P.O. Box 214, Ghana

**Keywords:** carbon nanodots, cannabidiol, drug delivery, evidence classification, nanocarriers, pharmacokinetics, safety, targeting

## Abstract

Cannabidiol (CBD) is a highly lipophilic phytocannabinoid whose low aqueous solubility, variable oral absorption, extensive first-pass metabolism, and chemical instability motivate advanced delivery strategies. Carbon nanodots (CNDs) have tunable surface chemistry and photoluminescence and have been investigated as carriers for multiple therapeutic cargos. This review critically evaluates whether the literature supports a specific CBD–CND delivery advantage. A structured narrative search of PubMed and targeted publisher websites was conducted. Evidence was classified as direct CBD–CND evidence, indirect CND-platform evidence, or indirect CBD-nanoformulation evidence. No direct study was identified that characterized CBD loading on CNDs and then demonstrated release, pharmacokinetics, safety, or therapeutic efficacy. Existing support is therefore indirect: CNDs can carry other drugs, whereas lipid, polymeric, micellar, and transdermal systems can improve selected CBD formulation or preclinical endpoints. Consequently, improved solubility, targeting, lower toxicity, enhanced bioavailability, and therapeutic synergy remain hypotheses for CBD–CND systems. The review retains a broad discussion of possible applications while clearly separating demonstrated evidence from proposed uses and defining the analytical, comparative, pharmacokinetic, biodistribution, and toxicological studies needed to validate the platform.

## 1. Introduction

Drug delivery is a vital aspect of modern healthcare and pharmaceutical science, playing a pivotal role in ensuring that therapeutic substances reach their intended targets in the body with precision and efficacy. In the context of carbon nanodots (CNDs) and cannabidiol (CBD), this review examines a proposed intersection between CBD nanocarrier research and CND-based therapeutic delivery [1,2]. No direct experimental CBD-loaded CND study meeting the formulation-characterization criteria defined in Section 3 was identified; therefore, CND-specific enhancement of CBD delivery, bioavailability, or therapeutic efficacy is not treated as an established outcome.

CBD, a non-psychoactive component of the cannabis plant, has attracted interest for potential therapeutic effects, although the strength of evidence varies by indication [3]. The challenge lies in delivering CBD in a controlled and effective manner because its low aqueous solubility, variable absorption, metabolism, and stability can limit exposure. Nanomedicine may address selected formulation endpoints, but any carrier-specific advantage must be measured directly [4].

Carbon nanodots (CNDs) are small carbon nanoparticles with optical properties, tunable surface chemistry, and a large surface-to-volume ratio. Selected CND formulations have transported non-CBD cargos, but those studies do not demonstrate CBD encapsulation, formulation-specific biocompatibility, or therapeutic benefit [5,6]. An earlier version of this review is available as a preprint [7]. A future CBD–CND formulation could be evaluated for loading, release, dispersion, targeting, pharmacokinetics, and biodistribution; none of these outcomes can be inferred solely from unrelated cargos.

## 2. Objectives and Scope

We seek to comprehensively review the potential of CNDs in enhancing the delivery and therapeutic efficacy of CBD. By collating and analyzing existing research and developments in this emerging field, we aim to elucidate the opportunities, challenges, and implications of integrating nanotechnology into CBD delivery. More specifically, the review aims to: (i) distinguish direct CBD–CND evidence from indirect platform and formulation evidence; (ii) evaluate candidate loading, release, targeting, safety, and therapeutic mechanisms; and (iii) define the experimental sequence required before a CBD–CND advantage can be claimed. The principal innovation is the explicit evidence-classification framework and critical comparison with contemporary CBD nanocarriers, rather than presentation of CNDs as an already validated CBD carrier.

## 3. Literature Search Strategy and Evidence Classification

A structured narrative search was conducted in PubMed and through targeted searches of publisher websites through 4 September 2026. Search terms combined ‘cannabidiol’ or ‘CBD’ with ‘carbon dots’, ‘carbon nanodots’, ‘carbon quantum dots’, ‘nanoparticle’, ‘drug delivery’, ‘loading’, ‘encapsulation’, ‘release’, ‘bioavailability’, ‘targeting’, ‘pharmacokinetics’, ‘toxicity’, and ‘biocompatibility’. Reference lists of relevant reviews and primary studies were also screened. English-language peer-reviewed articles addressing CBD formulation, CND drug delivery, CND safety, or targeting were prioritized; preprints were identified separately and were not treated as peer-reviewed evidence.

Evidence was classified as: (i) direct evidence, when CBD was experimentally loaded onto or into a characterized CND formulation and evaluated; (ii) indirect platform evidence, when CNDs carried another therapeutic cargo; or (iii) indirect CBD-formulation evidence, when CBD was delivered by a non-CND carrier. This was a structured narrative review rather than a registered systematic review or meta-analysis. No direct study was identified that completed the minimum characterization and biological comparison needed to establish CBD–CND synergy.

Studies were selected when they addressed at least one of the following: CBD physicochemical or pharmacokinetic limitations; CBD delivery using a defined nanocarrier; CND loading, release, targeting, or toxicity; or formulation-specific translational requirements. Studies were excluded from the core evidentiary assessment when the carrier or cargo was undefined, the publication was non-scholarly, or the findings could not be traced to an identifiable source. Because no direct CBD–CND formulation study met the definition above, indirect evidence was retained only to inform design principles and was explicitly labeled. Article selection was performed narratively; duplicate reports and preprints were separated from peer-reviewed evidence, and no formal risk-of-bias assessment or meta-analysis was undertaken.

## 4. Carbon Nanodots as Proposed Drug-Delivery Platforms

### 4.1. Physicochemical Properties and Synthesis

Carbon nanodots (CNDs), also called carbon quantum dots in parts of the literature, are carbon-based nanomaterials that are often below 10 nm before further functionalization [5]. Hydrothermal synthesis, microwave-assisted synthesis, and pyrolysis can produce different core structures, size distributions, heteroatom contents, and carboxyl, hydroxyl, or amino surface groups. These features influence aqueous dispersion, charge, ligand attachment, cargo association, and release; consequently, performance cannot be assigned to CNDs as a single uniform material. Representative primary carrier studies illustrate this formulation dependence (Table 1): microwave-pyrolyzed CNDs carrying curcumin were approximately 10 nm or smaller, hydrothermal hyaluronic acid/carboxymethyl chitosan CNDs were approximately 6 nm before doxorubicin loading, and a 1.5 nm nitrogen-doped CND system became a 154.7 nm functionalized nanocomposite [6,8,9]. None of these studies used CBD.

**Table 1:** Representative primary CND carrier studies showing synthesis- and surface-dependent formulation behavior; none used CBD as the cargo.

No.	CND Synthesis and Cargo	Measured Formulation Properties	Evidence Boundary and Design Implication
1	Microwave-pyrolyzed E-CND and citric acid/urea CND; curcumin	Curcumin–CND formulations were approximately 10 nm or smaller; adsorption efficiencies were 91% and 82%, loading capacity was 3.4–3.8 wt%, and release reached 60% and 74% at 72 h in pH 5 buffer and approximately 90% by 96 h in culture medium.	Primary CND carrier evidence, but the cargo was curcumin—not CBD. The results support measuring size, loading, and medium-dependent release rather than assuming transferability to CBD [6].
2	One-pot hydrothermal HA/CMCS CND; doxorubicin	The CNDs were approximately 6 nm and contained carboxyl, hydroxyl, and amino surface groups. Electrostatic doxorubicin loading produced a complex with an average AFM height of approximately 20 nm.	Surface chemistry and the loading mechanism were formulation-specific; neither CBD loading nor CBD release was studied [8].
3	Hydrothermal N-doped CND functionalized with glycyrrhizin/folate; doxorubicin	The starting CNDs averaged 1.5 nm with a zeta potential of −32.8 mV; after functionalization, the nanocomposite averaged 154.7 nm with a zeta potential of −44.6 mV. Doxorubicin was loaded through covalent and noncovalent interactions.	The large change after functionalization shows that final-product size and charge must be measured. This was not a CBD formulation [9].

### 4.2. Drug-Delivery Applications

Carbon nanodots have garnered attention as experimental drug-delivery platforms. Table 1 compares primary studies in which the synthesis route, starting size, surface chemistry, cargo, and quantitative formulation behavior were reported. The results show that loading and release depend on the exact CND preparation and surface modification. Because the cargos were curcumin or doxorubicin, the data provide design principles only and should not be interpreted as evidence that CNDs enhance CBD solubility, stability, bioavailability, or efficacy [6,8,9].

### 4.3. Drug Encapsulation and Surface Functionalization

The small size and modifiable surface of selected CND preparations can support cargo association and cellular uptake, but loading efficiency and biological compatibility are formulation dependent. Figure 1 illustrates an antibody-complexed CND concept for detecting a specific bacterial strain in serum; it is an analytical example rather than CBD-delivery evidence. Physical association, electrostatic loading, covalent conjugation, and ligand attachment have been demonstrated with other cargos [6,8,9]. For CBD, each proposed mechanism requires direct confirmation of loading capacity, chemical integrity, release, colloidal stability, cellular response, and target-specific uptake.

**Figure 1:**
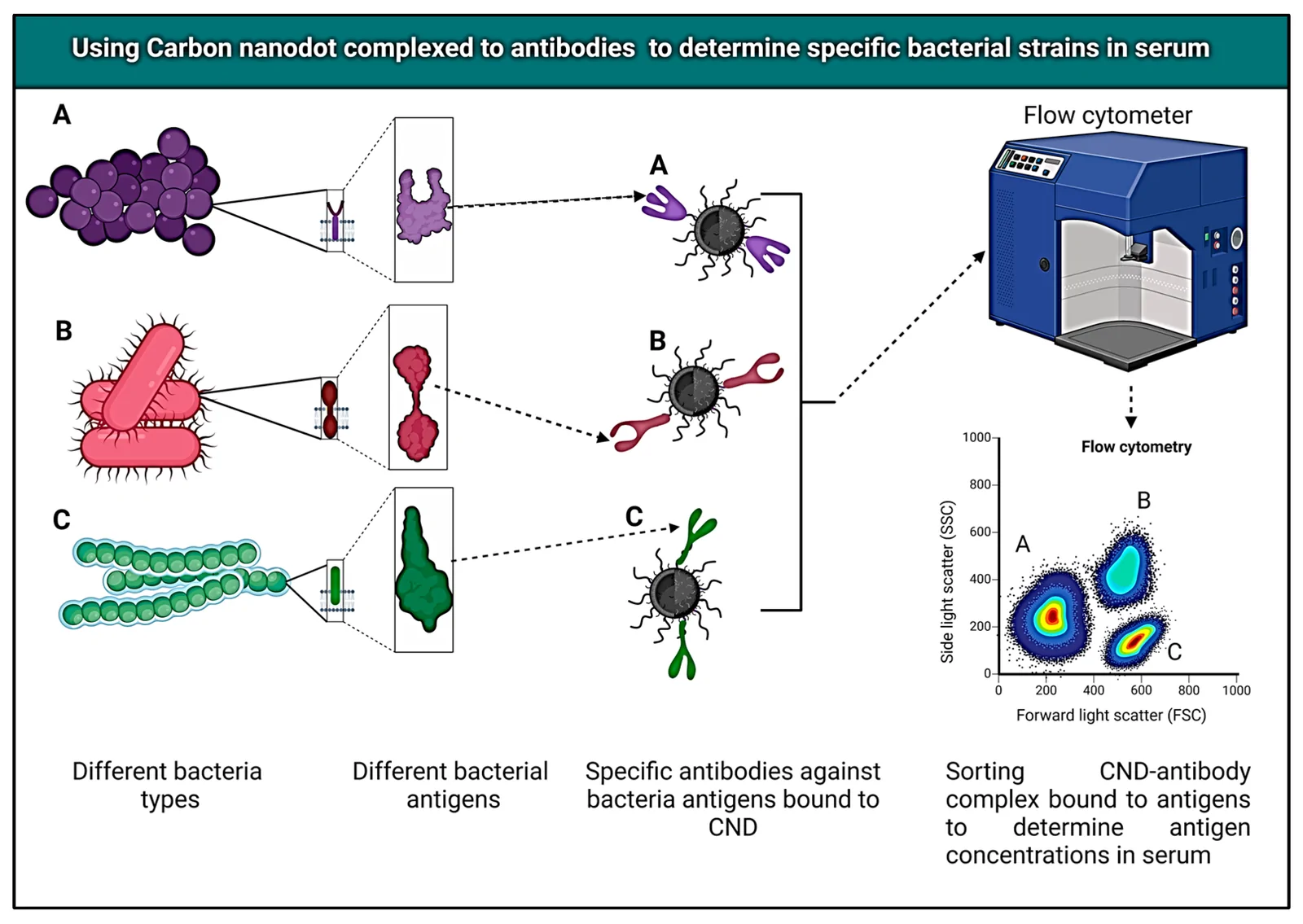
Using CNDs complexed with antibodies to determine specific bacterial strains in bacterially infected serum.

## 5. Cannabidiol: Therapeutic Context and Delivery Challenges

Cannabidiol (CBD), a naturally occurring phytocannabinoid described alongside other major cannabinoids in Table 2, is abundant in hemp and is non-intoxicating. CBD has been investigated for anti-inflammatory, analgesic, anxiolytic, anticonvulsant, and neuroprotective effects, but evidence strength and regulatory status differ substantially by indication [3,10]. Accordingly, this review separates established uses and experimentally observed CBD effects from proposed delivery applications.

### 5.1. Major Cannabinoids and Physicochemical Properties

Table 2 provides the molecular formula, molar mass, qualitative solubility, PubChem XLogP value, and chemical structure of selected cannabis-related compounds.

**Table 2:** Physicochemical properties of selected cannabis-related compounds.

No.	Compound	Molecular Formula	Molar Mass (g/mol)	Qualitative Solubility	XLogP (PubChem)	Structure
1	Δ9-Tetrahydrocannabinol (Δ9-THC) CID 16078	C_21_H_30_O_2_	314.5	Lipophilic; very low aqueous solubility; soluble in common organic and lipid vehicles.	7.0	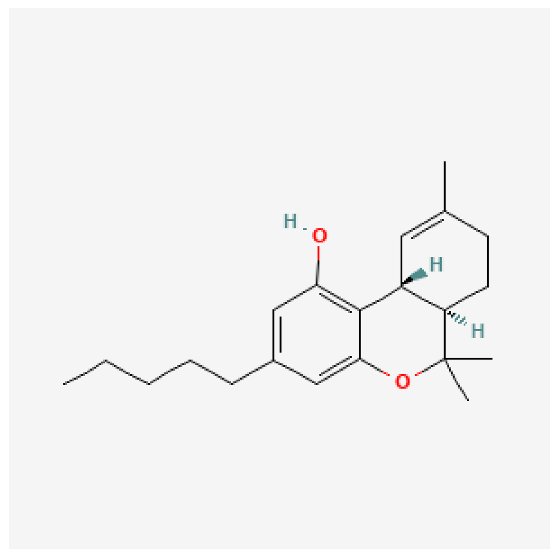
2	Cannabidiol (CBD) CID 644019	C_21_H_30_O_2_	314.5	Lipophilic; very low aqueous solubility; soluble in common organic and lipid vehicles.	6.5	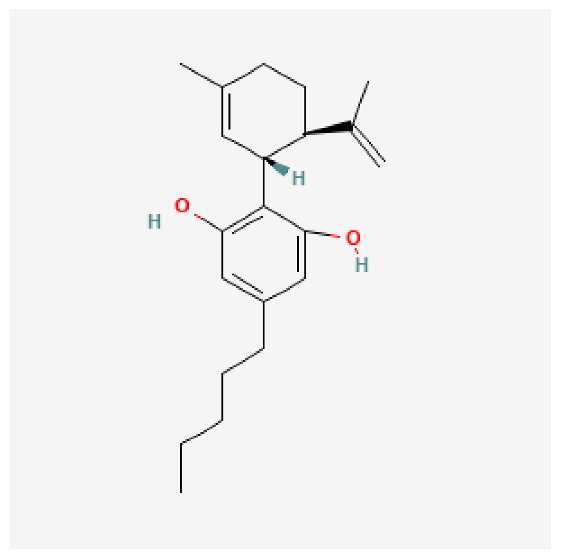
3	Cannabigerol (CBG) CID 5315659	C_21_H_32_O_2_	316.5	Lipophilic; very low aqueous solubility; soluble in common organic and lipid vehicles.	7.4	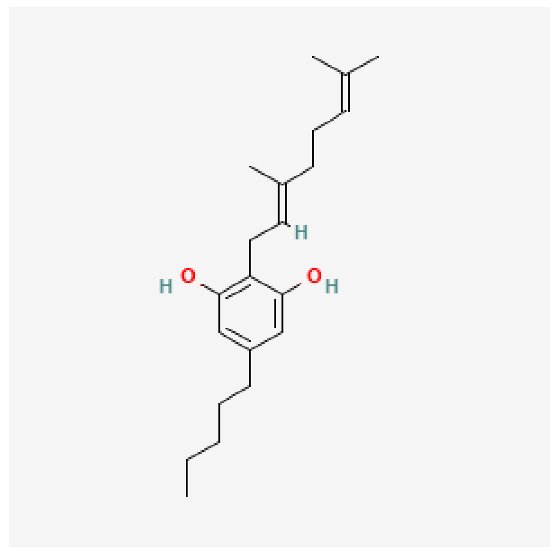
4	Cannabichromene (CBC) CID 30219	C_21_H_30_O_2_	314.5	Lipophilic; very low aqueous solubility; soluble in common organic and lipid vehicles.	6.9	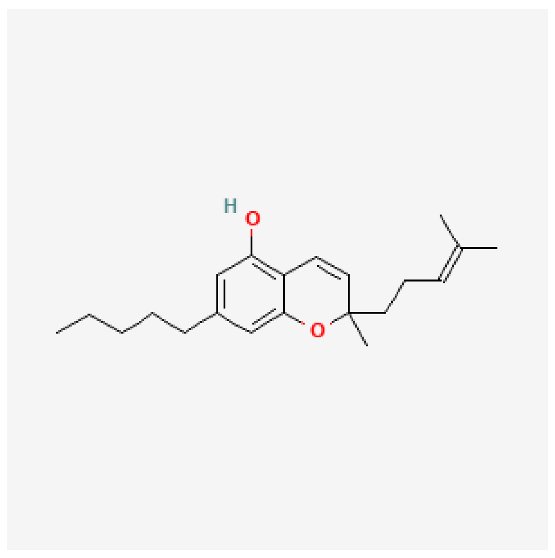
5	Cannabinol (CBN) CID 2543	C_21_H_26_O_2_	310.4	Lipophilic; very low aqueous solubility; soluble in common organic and lipid vehicles.	6.1	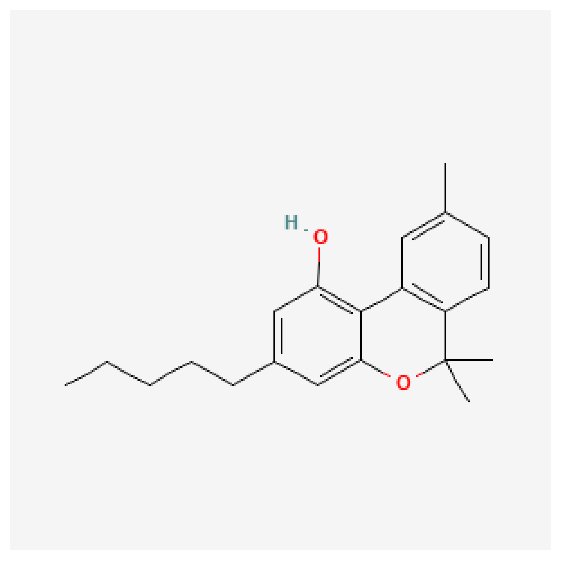
6	Δ9-Tetrahydrocannabinolic acid A (THCA-A) CID 98523	C_22_H_30_O_4_	358.5	Lipophilic acidic cannabinoid; low aqueous solubility; decarboxylates to Δ9-THC.	7.0	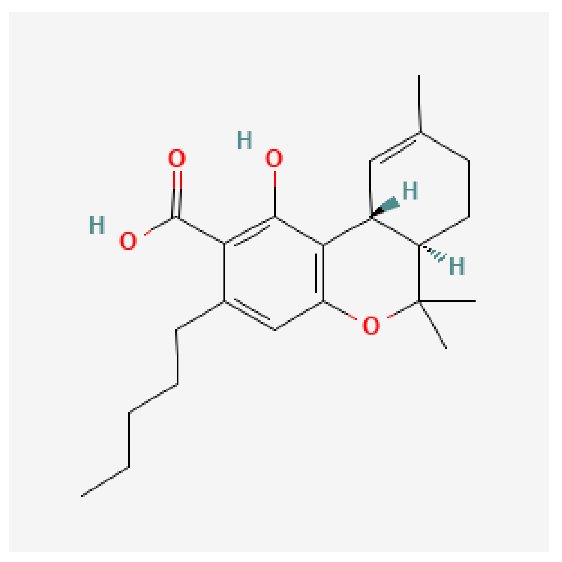
7	Cannabidiolic acid (CBDA) CID 160570	C_22_H_30_O_4_	358.5	Lipophilic acidic cannabinoid; low aqueous solubility; decarboxylates to CBD.	6.6	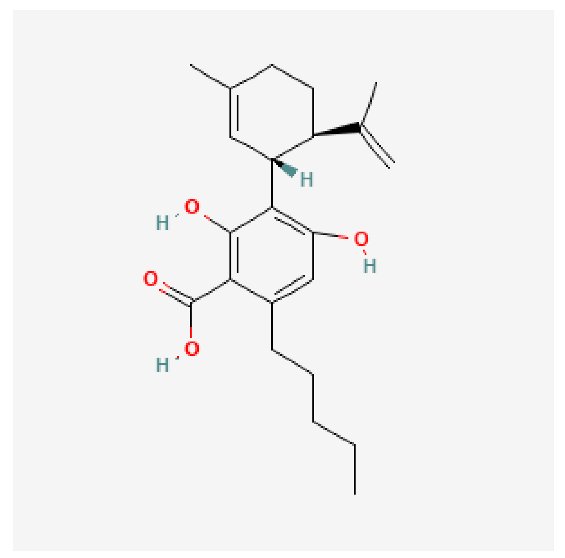
8	Cannabigerolic acid (CBGA) CID 6449999	C_22_H_32_O_4_	360.5	Lipophilic acidic cannabinoid; low aqueous solubility; biosynthetic precursor of major cannabinoids.	7.5	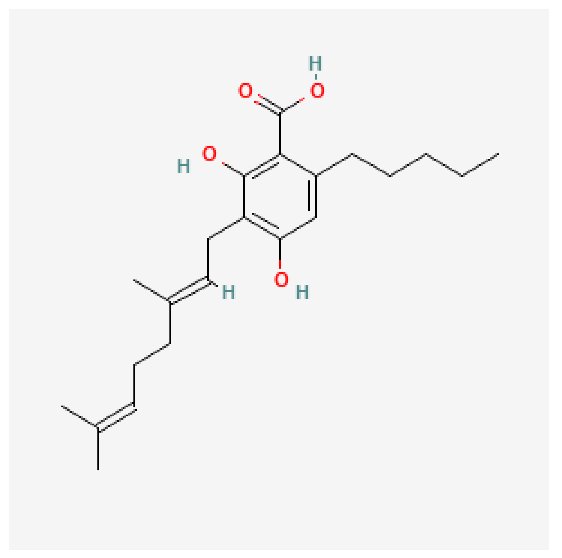
9	(6aR,9S)-Δ10-Tetrahydrocannabinol CID 101651232	C_21_H_30_O_2_	314.5	Lipophilic; very low aqueous solubility; compound identity follows the cited PubChem record.	6.0	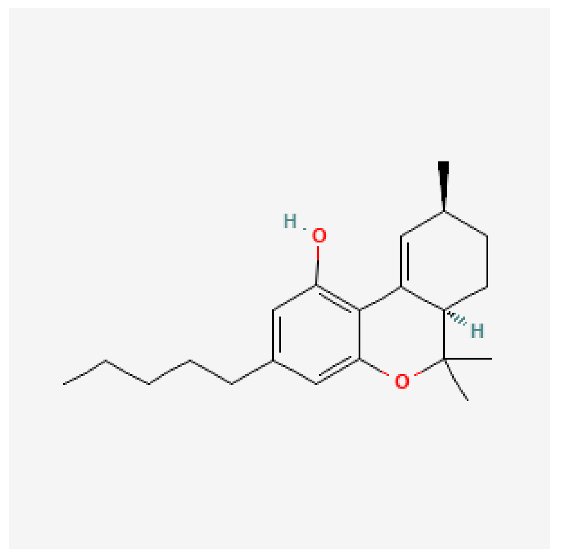
10	Δ8-Tetrahydrocannabinol (Δ8-THC) CID 638026	C_21_H_30_O_2_	314.5	Lipophilic; very low aqueous solubility; compound identity follows the cited PubChem record.	5.7	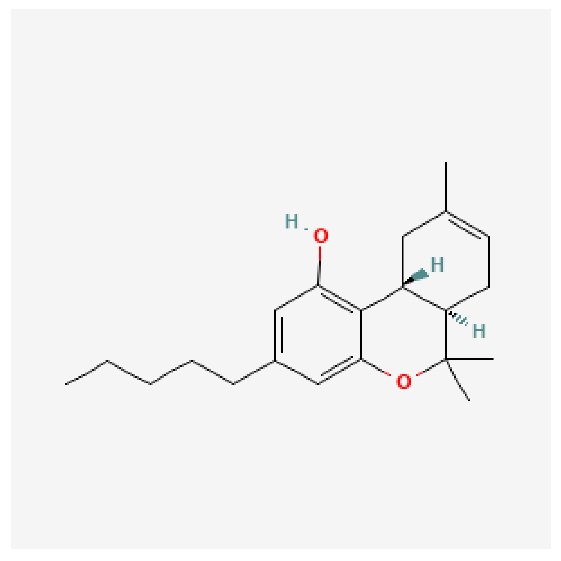

Note: XLogP values are PubChem predictions rather than experimentally measured logP values. Compound-specific CIDs are provided in the table, with direct record URLs in reference [11]. Structures, molecular weights, and XLogP values were verified against these records on 23 September 2026 [11,12]. Molecular weights follow the precision reported by PubChem. The THCA-A entry uses CID 98523; the Δ10-THC entry specifically represents the (6aR,9S) stereoisomer, CID 101651232.

### 5.2. Therapeutic Mechanisms and Clinical Context

CBD interacts with several molecular targets, including components of endocannabinoid-related signaling, but the relevance of an individual pathway depends on dose, model, and indication [3,10]. Clinical evidence is established for purified CBD in specific seizure disorders; broader claims in pain, anxiety, and neurodegenerative disease remain indication and formulation dependent. Figure 2 summarizes proposed antiepileptic actions involving intracellular calcium signaling, GPR55, TRPV1, adenosine uptake, and synaptic transmission [13,14,15]. These CBD mechanisms do not by themselves demonstrate that a CND carrier improves treatment.

**Figure 2:**
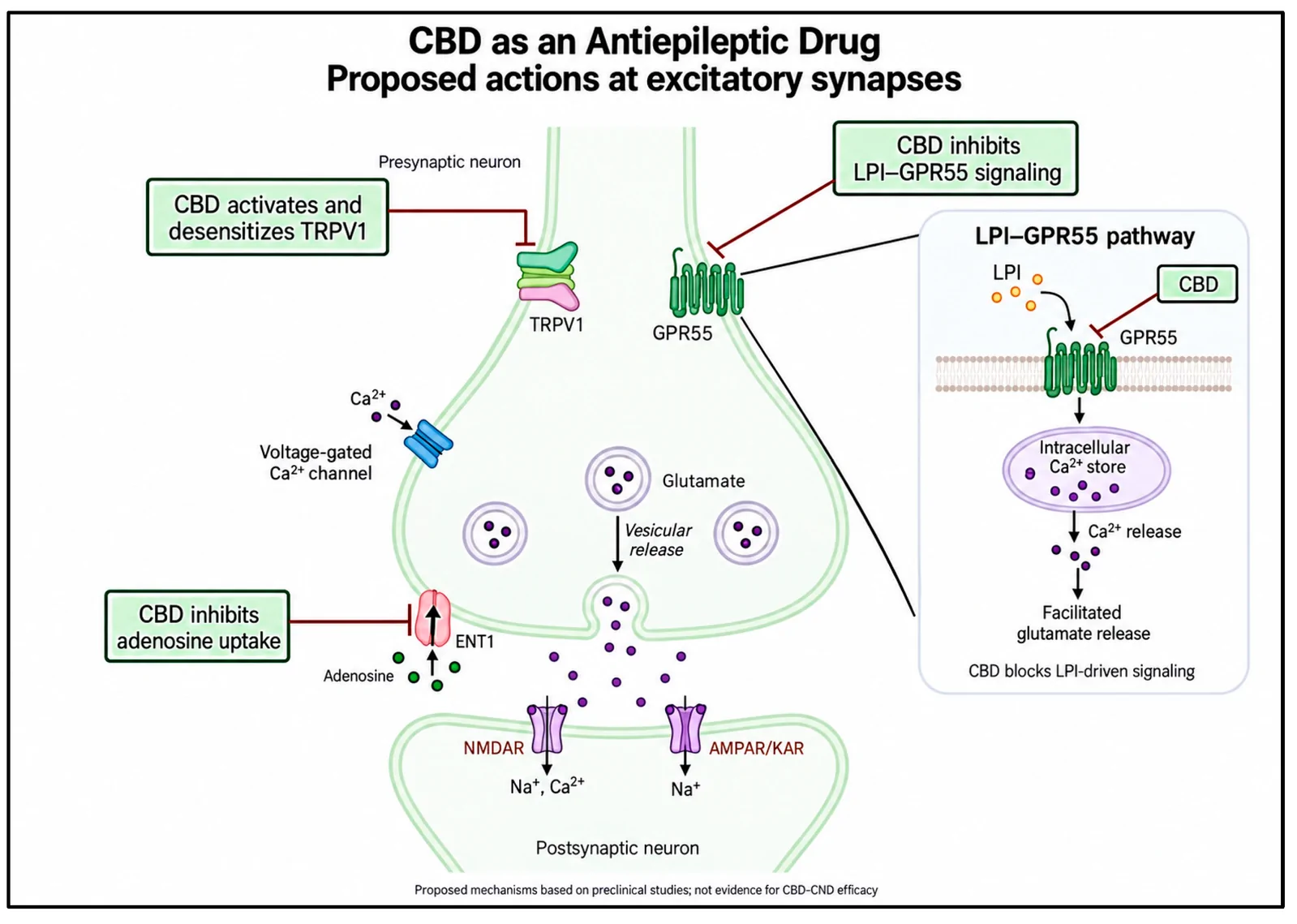
Proposed actions of CBD as an antiepileptic drug at excitatory synapses. CBD can inhibit LPI–GPR55 signaling associated with calcium release from intracellular stores and enhanced glutamate release [13]. Other preclinical findings include TRPV1 activation followed by desensitization [14] and inhibition of ENT1-mediated adenosine uptake [15]. These findings arise from different experimental systems and do not establish a complete clinical mechanism or demonstrate improved efficacy from CND delivery. GPR55 is depicted as a G-protein-coupled receptor, distinct from the voltage-gated calcium channel; cAMP, GIRK, and SNARE effects are not assigned to GPR55.

### 5.3. Current Delivery Challenges

Evidence boundary: the therapeutic applications discussed below are prospective research directions. No direct CBD-loaded CND efficacy study was identified; statements concerning improved delivery or clinical benefit must therefore be read as hypotheses requiring experimental validation. However, as with many promising agents, the efficacy of CBD hinges significantly on the art and science of drug delivery. Some of the most pressing challenges stem from the route of administration. When administered orally, CBD grapples with poor bioavailability, signifying that a substantial portion of the compound gets lost in the convoluted processes of digestion and liver metabolism. This leads to suboptimal outcomes in terms of the actual dose of CBD that the body can absorb and utilize. The variability in accurate and consistent dosing, especially when using conventional delivery methods, remains a formidable obstacle. This challenge is paramount in medical applications, where precision is vital [4,16]. Another intricate facet of CBD delivery is the timing of therapeutic effects. It is not just about what CBD can do but also about how quickly it can do it.

The onset of action varies with the delivery route and formulation. CBD can also be sensitive to light, temperature, oxygen, and matrix conditions, so preservation of chemical integrity from production to administration is important [17,18]. For conditions requiring local exposure, an advanced carrier may be useful, but improved localization or therapeutic outcome must be demonstrated against free CBD and an appropriate benchmark formulation. Formulation advantages such as higher apparent solubility or slower in vitro release do not automatically establish greater clinical efficacy [19].

## 6. Proposed Therapeutic Applications of CBD–CND Systems

### 6.1. Pain Management

Pain management is a critical aspect of healthcare, and a CBD-loaded CND formulation could be investigated for pain-related conditions; it is not yet a demonstrated treatment. Medical cannabis and cannabinoids have been evaluated for chronic non-cancer and cancer-related pain, but the reported benefits are generally small and evidence is indication-specific [20]. CBD has also been incorporated into nanoemulsions and nanoemulsion-filled hydrogels, although that formulation study did not demonstrate pain relief or a CND effect [21]. Limited aqueous solubility and variable bioavailability complicate consistent CBD exposure. A CBD–CND system would require direct evidence of loading, stability, pharmacokinetics, tissue exposure, and superior pain-related outcomes before any advantage could be claimed.

Whether CNDs can deliver CBD efficiently to neural tissues or improve neuropathic pain outcomes remains untested [19]. Patients undergoing cancer treatments often experience severe pain, which can significantly affect their quality of life. CBD has been explored for its potential to manage cancer-related pain due to its analgesic and anti-inflammatory effects. Enhanced effectiveness has not yet been demonstrated for CBD delivered by CNDs. If validated for a specific CBD–CND formulation, sustained release could reduce dosing frequency; prolonged pain relief and fewer adverse effects have not been demonstrated [6]. To establish the efficacy of CBD-loaded CNDs in pain management, comprehensive clinical studies are essential. These studies should focus on specific pain conditions, dosing regimens, and patient populations. Additionally, long-term safety assessments are critical to ensure that the therapy remains well tolerated over extended periods. Collaborations between researchers, pharmaceutical companies, and healthcare providers are necessary to drive these studies and translate the potential of this innovative drug delivery system into tangible pain relief solutions for patients.

### 6.2. Neurological Disorders

Neurological disorders represent a diverse group of conditions that affect the nervous system and can have a profound impact on an individual’s quality of life. CBD is being investigated for neuroprotective effects, but whether a CND formulation can improve its delivery for neurological conditions remains an experimental question. The cited CND literature concerns synthesis, bioelectrical applications, cell-growth studies, antioxidant behavior, or bioimaging rather than CBD delivery to the nervous system [22,23,24]. Epilepsy is characterized by recurrent seizures caused by abnormal electrical activity in the brain. Randomized trials have shown that purified CBD can reduce seizures in Dravet syndrome and drop seizures in Lennox–Gastaut syndrome [25,26]. However, dosage and consistency of CBD administration are crucial for efficacy. CNDs could be investigated as one possible delivery platform, but brain exposure, dose consistency, and comparative efficacy require direct measurement. Whether this approach improves seizure control or quality of life remains untested. Multiple sclerosis is an autoimmune disease that affects the central nervous system.

It leads to a range of symptoms, including muscle spasms, pain, and difficulties with mobility, while oxidative and inflammatory pathways contribute to disease pathology [27]. CBD has anti-inflammatory activity in the experimental literature, but its value for managing multiple sclerosis symptoms cannot be inferred from those mechanisms alone [28,29]. CND-enabled delivery to target areas of the central nervous system is a hypothesis that requires biodistribution and efficacy testing. Neurodegenerative disorders such as Alzheimer’s and Parkinson’s disease are characterized by progressive neuronal dysfunction and loss [30]. CBD’s potential neuroprotective and anti-neuroinflammatory effects have generated interest in these conditions [29]. It remains to be determined whether a CND formulation can improve CBD stability, brain exposure, or neuroprotective activity. To evaluate CBD-loaded CNDs in neurological disorders, controlled studies must first establish the formulation, dose, brain distribution, safety, and comparative efficacy before clinical trials are considered.

### 6.3. Inflammatory Disorders

Inflammatory disorders encompass conditions in which persistent immune activation contributes to pain and tissue injury [31,32]. CBD has shown anti-inflammatory effects in experimental models, but the findings do not establish effectiveness across rheumatoid arthritis, inflammatory bowel disease, or inflammatory skin disease [29]. CND-mediated delivery of CBD to inflamed joint, intestinal, or skin tissue has not been demonstrated. Any proposed benefit must therefore be evaluated through formulation-specific biodistribution and comparative efficacy studies rather than inferred from CBD activity alone.

A general CND review describes synthesis and applications [33], but it does not establish that CNDs improve CBD bioavailability or delivery to inflamed intestinal tissue. If such delivery is demonstrated, its effects on inflammation, pain, and gastrointestinal function should then be evaluated. Inflammatory skin disorders such as psoriasis and eczema involve persistent skin inflammation and disruption of local immune or barrier processes [34,35]. CBD’s anti-inflammatory properties have prompted investigation of topical use [36,37]. A murine study demonstrated transdermal CBD delivery and an anti-inflammatory effect [38], but a CBD–CND topical formulation would require separate measurements of dermal penetration, local exposure, efficacy, and safety. Clinical validation would then be necessary to define indication, dose, and benefit.

### 6.4. Oncology, Mental Health, and Emerging Directions

Oncology and mental health applications are proposed research directions. Reviews describe CBD activity and symptom-management questions in cancer, but they do not establish a CBD–CND treatment [39,40]. Figure 3 therefore presents peptide-targeted CBD–CND delivery only as a conceptual workflow; receptor-dependent uptake, tumor exposure, antitumor activity, symptom relief, and safety require direct controls. CBD has also been studied in anxiety- and depression-related models [41,42], yet no evidence shows that CND loading improves psychiatric outcomes, dosing precision, onset, adherence, or tolerability.

**Figure 3:**
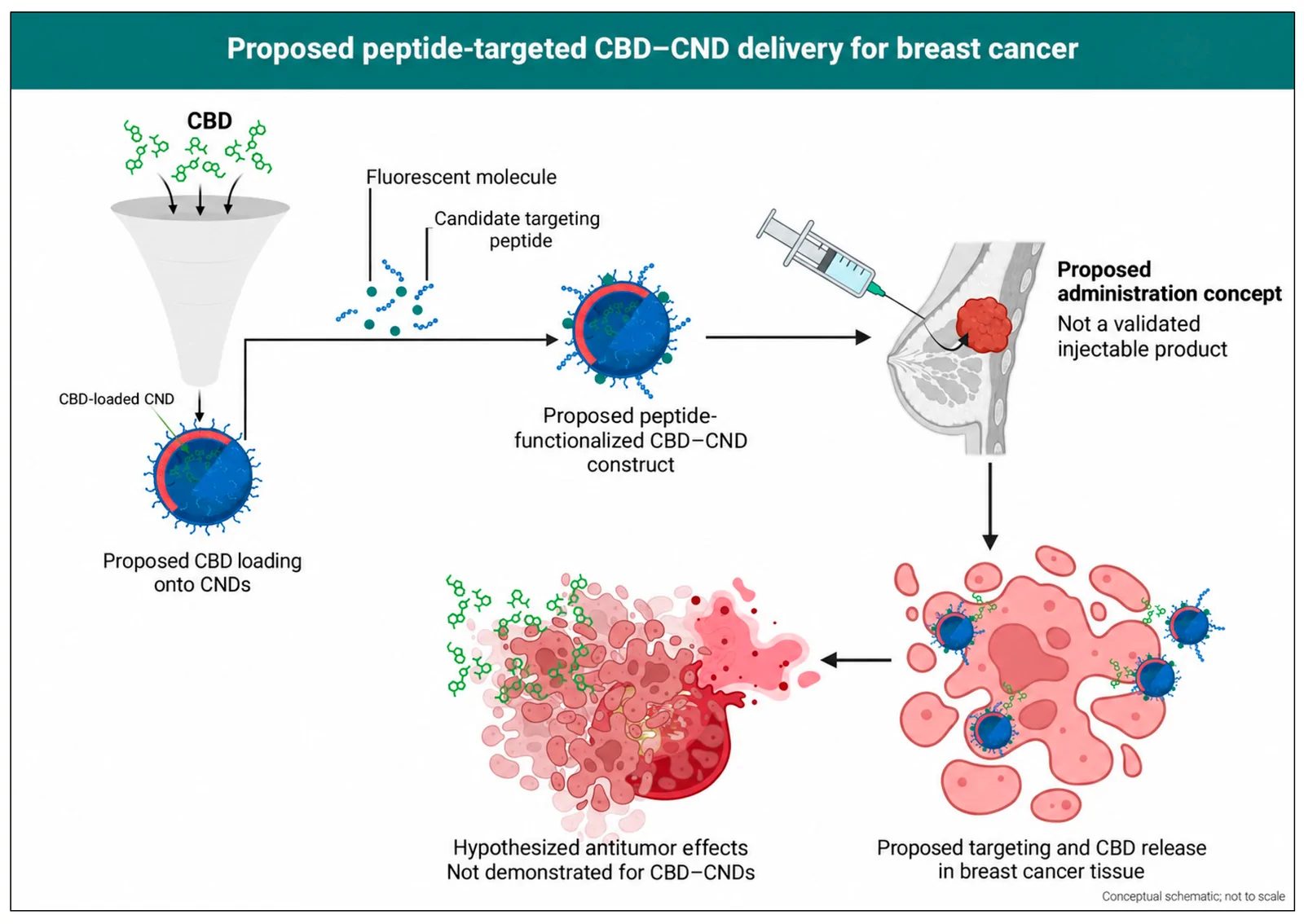
Conceptual workflow for a proposed peptide-targeted CBD–CND formulation in breast cancer. The schematic illustrates candidate CBD loading, peptide functionalization, receptor-dependent uptake, and local release as hypotheses only. It does not represent an injectable clinical product or demonstrate tumor targeting, anticancer efficacy, pharmacokinetics, biodistribution, or safety. Each step requires formulation characterization and appropriate in vitro and in vivo controls before clinical use could be considered.

### 6.5. Cross-Application Critical Synthesis

Across these indications, the limiting step is not the absence of plausible biological targets for CBD; it is the absence of formulation-specific evidence showing that a CND carrier alters CBD exposure or therapeutic performance. Pain, neurological, inflammatory, and oncology applications therefore should not be ranked by disease appeal. They should be prioritized according to measurable formulation attributes, target-tissue exposure, clinically relevant comparators, and safety margins. Neurological applications additionally require brain biodistribution, topical inflammatory applications require dermal penetration, and oncology applications require receptor-dependent uptake and comparative tumor-to-healthy-tissue distribution. This cross-domain comparison prevents evidence from one route or disease model from being transferred uncritically to another.

## 7. Quantitative CBD Nanodelivery Evidence

Recent work confirms that CBD formulation remains an active field. CBD-loaded nanostructured lipid carriers, a micellar/vesicular transdermal patch, and nanosuspension-loaded dissolving microneedles have produced measurable formulation or pharmacokinetic results [43,44,45]. Functionalized lipid nanoparticles, intranasal PLGA nanoparticles, and polymeric nanomicelles have also been investigated for BBB transport, brain-targeted delivery, or oral delivery [46,47,48]. Every system in this paragraph is a non-CND comparator.

Oncology-oriented studies have examined folate–chitosan nanoparticles carrying CBD and lipid nanoparticle co-delivery of CBD with paclitaxel [49,50]. Recent reviews further emphasize both the promise and formulation-specific limitations of cannabinoid nanodelivery [51,52,53]. These studies provide useful comparators and design principles, but none uses CNDs as the CBD carrier.

Table 3 summarizes the CBD-specific quantitative evidence used in this review. The three experimental systems are lipid, micellar/vesicular, or nanosuspension-based carriers rather than CNDs. They demonstrate which formulation and pharmacokinetic parameters can be measured, but they do not establish that a CND carrier will produce the same results.

**Table 3:** Quantitative primary evidence for CBD nanodelivery and the boundary of its relevance to proposed CBD–CND systems.

Study/System	Carrier and Formulation	Quantitative Result	Evidence Boundary
Xie et al., 2024 [43]	CBD-loaded nanostructured lipid carrier	Mean size 54.33 nm; PDI 0.118; zeta potential −29.7 mV; encapsulation efficiency 87.58%; >50% release within 20 min followed by gradual release; reduced photodegradation and stability reported for 42 days.	CBD-specific primary formulation and stability evidence, but the carrier was not a CND, and no AUC or Cmax was reported.
Chu et al., 2024 [44]	CBD-loaded micellar/vesicular transdermal nanocarrier patch	Selected transdermal delivery measures were 3.68 ± 0.17-fold and 2.46 ± 0.18-fold versus free CBD comparators; rat pharmacokinetic relative bioavailability was 9.47 ± 0.88-fold versus oral CBD.	CBD-specific non-CND evidence; the formulation, route, and comparator prevent attribution of these gains to CNDs.
Cheng et al., 2024 [45]	CBD nanosuspension-loaded dissolving microneedle patch	In rats, Cmax was 144.4 ± 39.4 ng/mL, Tmax was 12 h, and AUC0–∞ was 2.47-fold that of oral CBD; concentrations remained >10 ng/mL for up to 72 h after patch removal at 24 h.	CBD-specific preclinical non-CND evidence; these data do not demonstrate a CND effect or clinical efficacy.

## 8. Critical Appraisal of CBD–CND Evidence

No direct CBD–CND study was identified that simultaneously established formulation composition, CBD loading, release kinetics, stability, pharmacokinetics, biodistribution, safety, and therapeutic performance. Accordingly, the broad applications retained in the following sections describe a research agenda rather than validated CBD–CND products.

In this review, ‘synergy’ is reserved for a quantitatively greater-than-additive effect tested with an appropriate reference model. A formulation that outperforms free CBD may demonstrate a delivery advantage, but that result alone is not evidence of synergy. Future studies should compare free CBD, unloaded CNDs, a physical mixture, CBD–CNDs, and a benchmark carrier at matched CBD and nanomaterial doses.

## 9. Emerging Research Directions

### Cardiovascular, Precision Medicine, and Neuropharmacology Concepts

The applications and mechanisms in this section are proposed possibilities, not established outcomes for CBD–CND formulations. CBD supplementation has been associated with blood pressure changes in a specific human study [54], but this is CBD evidence and does not demonstrate a cardiovascular benefit from CND delivery. Precision dosing with a CBD–CND product remains a formulation concept. Likewise, evidence concerning CBD in neurological or psychiatric contexts and CBD nanoparticles or nanoemulsions in the central nervous system involves CBD alone or non-CND carriers [55,56]. Cardiovascular protection, stroke prevention, psychiatric efficacy, and patient-specific delivery must not be attributed to CBD–CNDs without direct pharmacokinetic, biodistribution, and disease-model comparisons.

Translational research is necessary to determine whether the proposed CBD–CND concept can progress beyond laboratory formulation studies. Collaboration among formulation scientists, toxicologists, pharmacologists, clinicians, and regulatory experts would be needed to define clinically relevant comparators and endpoints. Patient needs and route-specific constraints should guide development, but clinical positioning is premature until formulation identity, exposure, efficacy, and safety are demonstrated.

## 10. Proposed Integration of CNDs and CBD

Combining CNDs and CBD is a formulation hypothesis. This section therefore discusses candidate interactions, compatibility tests, and possible effects on solubility and bioavailability as questions to be evaluated rather than outcomes already achieved.

### 10.1. Proposed Mechanisms of Interaction

The proposed interaction between CNDs and CBD offers testable drug-delivery mechanisms, but enhancement has not been demonstrated experimentally. Quantitative evidence from another cargo illustrates the distinction: curcumin-loaded CNDs of approximately 10 nm or smaller showed adsorption efficiencies of 91% and 82%, loading capacities of 3.4–3.8 wt%, and medium-dependent release reaching 60% and 74% at 72 h in pH 5 buffer and approximately 90% by 96 h in culture medium [6]. These findings show that a defined CND formulation can carry and release curcumin; they do not establish CBD loading, protection, controlled release, bioavailability, or therapeutic efficacy. A CBD–CND study must independently measure association, chemical integrity, release kinetics, colloidal stability, pharmacokinetics, biodistribution, and biological activity.

For the compatibility and loading discussion below, the proposed effects on loading, release, safety, and bioavailability remain formulation hypotheses rather than direct findings from an experimentally characterized CBD–CND system.

### 10.2. Compatibility and Validation Requirements

Compatibility between a specific CND preparation and CBD cannot be assumed. CND composition, purification, surface chemistry, size, charge, dose, and route influence biological response, while CBD association may alter both colloidal behavior and drug availability. Functional groups can be selected to test noncovalent association or covalent attachment, but successful loading must be confirmed by orthogonal analytical methods and a mass balance. Absence of precipitation is not proof of chemical compatibility, and results from unloaded CNDs cannot establish the safety of a CBD–CND formulation. Formulation-specific cytotoxicity, genotoxicity, immune response, hemocompatibility, pharmacokinetics, biodistribution, and repeated-dose toxicity are therefore required.

### 10.3. Effects on Solubility and Bioavailability

Solubility and bioavailability are related but distinct endpoints. Table 3 provides CBD-specific quantitative results from three non-CND systems, including particle size, encapsulation efficiency, release, stability, Cmax, Tmax, AUC, and relative bioavailability [43,44,45]. These measurements show that CBD delivery can be modified by particular lipid, micellar/vesicular, or nanosuspension formulations. They do not show that CNDs act as CBD solubilizers or protective carriers, and the carbon-nanodot synthesis review [33] does not supply CBD formulation or pharmacokinetic evidence.

For a proposed CBD–CND system, equilibrium solubility or apparent solubility should be distinguished from temporary dispersion, and encapsulation efficiency should be reported together with loading capacity and recovery. Stability studies should quantify CBD and degradants under controlled light, temperature, oxygen, humidity, and matrix conditions. Pharmacokinetic comparison should report dose-normalized Cmax, Tmax, AUC, terminal half-life, and relative bioavailability against free CBD and a relevant established carrier. Until such data are available, protection, improved absorption, predictable exposure, and therapeutic advantage remain untested hypotheses.

### 10.4. Candidate CBD-Loading Strategies

Loading CBD onto CNDs is a proposed drug-delivery strategy. Candidate methods include noncovalent association or adsorption, incorporation during formation of a defined carbonaceous carrier, and covalent attachment through a validated linker. Studies with curcumin and doxorubicin show that CND synthesis and surface functionalization can influence size, charge, loading, and release [6,8,9], but those observations cannot be transferred directly to CBD. Each method must preserve CBD identity and biological activity and must be compared using consistent definitions of encapsulation efficiency and loading capacity.

#### 10.4.1. Physical Encapsulation

Physical incorporation or noncovalent association can be screened without derivatizing CBD, but the mechanism and location of the drug must be demonstrated rather than described as encapsulation by default. In the curcumin CND study summarized in Table 1, adsorption efficiency, loading capacity, particle size, and release were measured directly [6]. For CBD, comparable evidence would require recovery of unbound drug, orthogonal confirmation of association, size and zeta-potential measurements before and after loading, release testing under sink and non-sink conditions, and chemical-stability analysis.

Release may be altered by CND precursor, synthesis conditions, surface functional groups, pH, ionic strength, protein adsorption, and CBD-to-CND ratio. A slower release profile is not automatically preferable and should be linked to the intended route and therapeutic exposure. Burst release, incomplete recovery, membrane binding, or poor sink conditions can distort in vitro results, so free CBD and benchmark-carrier controls are necessary.

#### 10.4.2. Covalent Attachment

Covalent attachment of CBD to a CND is a proposed prodrug-like approach rather than an established encapsulation method. A linker would need to attach at a defined site without destroying the relevant CBD activity and then release intact CBD at an appropriate rate, unless the conjugate itself is the intended active entity. A doxorubicin example used 1.5 nm nitrogen-doped CNDs with glycyrrhizin and folate and produced a 154.7 nm functionalized nanocomposite with a zeta potential of −44.6 mV [9]. This non-CBD result demonstrates why the final conjugate—not only the starting CND—must be characterized. For CBD, bond identity, substitution ratio, free-drug contamination, linker cleavage, metabolites, and comparative activity would all require direct measurement.

#### 10.4.3. Adsorption

Adsorption is a candidate CBD-loading mechanism, but direct CBD–CND adsorption capacity and release have not been established. Cortés et al. used carbon xerogel microspheres (CXMS), not carbon nanodots, for delayed CBD release and subsequent aflatoxin adsorption [57]. That study is a carbonaceous-material comparator and does not demonstrate a CND formulation, nanoscale biodistribution, improved CBD bioavailability, or in vivo therapeutic efficacy. Its carrier identity and experimental scope are stated explicitly to prevent transfer of the findings to CBD–CNDs.

Adsorption may offer a relatively simple preparation route, but capacity and reversibility depend on surface area, pore structure, surface chemistry, solvent, ionic strength, pH, and CBD concentration. High loading cannot be assumed. A candidate CBD–CND system should report an adsorption isotherm where appropriate, loading capacity, encapsulation efficiency, desorption or release kinetics, mass balance, and retention of CBD integrity. Physical encapsulation, covalent attachment, and adsorption should then be compared at matched CBD and CND doses. Evidence from curcumin-loaded CNDs, CBD in other carriers, or CBD on carbon xerogel microspheres should remain identified as indirect [6,57,58,59].

## 11. Tissue-Specific Functionalization and Targeting

Surface decoration of CNDs has been explored in non-CBD imaging and therapeutic systems. Direct CND examples include AS1411 aptamer conjugation for cancer-cell detection and imaging [60,61], folate-functionalized fluorescent carbon nanoparticles [62], nucleolin-targeted ratiometric carbon dots [63], and sulforaphane-conjugated carbon dots for EGFR-overexpressing cells [64]. Antibody and aptamer strategies are also described in broader carbon-nanomaterial and nanoparticle literature [65,66,67]. These studies show that functionalization is technically possible for particular formulations; they do not establish tissue targeting, clinical precision, or therapeutic benefit for a CBD-loaded CND. The final conjugate must be characterized for ligand density, size, charge, binding specificity, stability, biodistribution, and off-target uptake.

Table 4 lists candidate cancer-targeting molecules and their associated biomarkers. The examples come from broader targeting literature and are included to guide experimental design. For a CBD–CND formulation, marker selection would require confirmation of receptor expression, ligand accessibility after conjugation, receptor-dependent uptake, competition controls, and comparison with an untargeted formulation. Diagnostic or surgical-guidance claims from other CND systems should not be transferred to CBD delivery.

Tissue-specific markers are intended to increase the proportion of a carrier reaching receptor-positive cells while limiting nonspecific exposure. For decorated CBD–CNDs, that outcome remains hypothetical. Binding to an isolated receptor is insufficient: studies must compare receptor-positive and receptor-negative cells, measure uptake and intracellular release, quantify tumor-to-healthy-tissue distribution, and determine whether any exposure change improves efficacy without increasing toxicity. Marker functionalization can also alter size, charge, clearance, and immune recognition, so the final formulation requires complete characterization.

### 11.1. Targeted Drug Delivery

The preceding discussion summarizes proposed benefits and limitations of marker-decorated CNDs. Direct studies of CNDs without CBD demonstrate specific diagnostic, imaging, or theranostic constructs [60,61,62,63,64,65,68,69], while the targeting strategies summarized in Table 4 arise mainly from broader antibody, ligand, liposome, and nanoparticle literature [70,71,72,73,74,75,76,77,78,79,80,81,82,83,84,85]. All of this evidence remains indirect for CBD–CNDs, and formulation-specific binding, biodistribution, efficacy, and safety must be demonstrated.

### 11.2. Candidate Targeting Molecules

Table 4 summarizes candidate targeting molecules, their associated biomarkers, and reported application contexts in the broader cancer-delivery literature. Their relevance to CBD–CND formulations remains to be established.

**Table 4:** Candidate targeting strategies from broader cancer-delivery literature; none establishes targeting by a CBD-loaded CND.

Targeting Strategy	Validated Target or Biomarker	Reported Application Context	Refs
Antibody–drug conjugates and monoclonal antibodies	HER2 and lymphoma-associated antigens	Gastric cancer and non-Hodgkin lymphoma	[70,71]
Hyaluronic acid	CD44	CD44-positive tumor models	[72,75,80]
Aptamers	Experimentally selected cell-surface targets	Cell recognition and detection	[73]
RGD peptides	Integrin αvβ5	Integrin-positive tumor models	[74]
Folic acid	Folate receptor α	FRα-positive tumors	[76]
Transferrin	Transferrin receptor	TfR-positive cancers and brain-delivery models	[77,85]
Anti-GD2 antibodies	GD2	High-risk neuroblastoma	[78]
CD133/EpCAM-directed ligands	CD133 and EpCAM	Liver cancer stem-cell models	[79,84]
Glycyrrhetinic acid	Hepatic glycyrrhetinic acid-binding sites	Liver cancer delivery models	[81]
PSMA ligands	Prostate-specific membrane antigen	Prostate cancer imaging and therapy	[82,83]

The molecules and receptors summarized above are candidate targeting strategies. Their usefulness for a CBD–CND formulation has not been demonstrated. These targeting molecules are selected based on the specific biomarkers or receptors overexpressed on cancer cells and are intended to improve delivery of drugs to the selected cancer sites.

### 11.3. Imaging and Diagnosis

Evidence for CND-based imaging and diagnosis comes from specific non-CBD systems. Motaghi et al. developed an AS1411 aptamer–carbon-dot construct for spectrofluorometric detection of cancer cells [60], and Kong et al. reported AS1411-modified carbon dots for targeted cancer-cell imaging [61]. Anbalagan et al. used biofunctionalized carbon dots in an electrochemical carcinoembryonic antigen biosensor [65], while Gutiérrez-Gálvez et al. reported a carbon-nanodot electrogenerated-chemiluminescence biosensor for miRNA-21 [68]. These studies establish particular assay designs and measured readouts; they do not demonstrate that decorated CNDs can diagnose any disease or deliver CBD.

Microfluidic diagnostic platforms provide useful context for early detection and personalized treatment, but the cited review is not evidence for a CND formulation [86]. Wang et al. developed multifunctional polymer-coated carbon nanodots for glioma-targeted theranostics [69], whereas transferrin-targeted liposomes are a non-CND comparator for brain delivery [85]. These examples do not establish real-time surgical guidance, treatment monitoring, or clinical diagnostic performance for a CBD–CND formulation. Such claims would require validated sensitivity, specificity, tissue penetration, target-to-background ratios, and comparison with current diagnostic standards.

Table 5 is therefore restricted to traceable examples of CND or closely related carbon-nanoparticle diagnostic constructs. Reviews of carbon dots in cancer nanomedicine and bioanalysis provide additional context [87,88], and aptamer-conjugated carbon nanomaterials have been reviewed for cancer and bacterial theranostics [67]. None of these systems contained CBD. Their relevance is limited to showing how a marker, target, readout, and validation strategy can be defined for a future CBD–CND study.

**Table 5:** Traceable diagnostic examples involving CNDs and related carbon nanoparticles without CBD.

No.	Marker or Construct	Demonstrated Non-CBD Application and Measured Target/Readout	Refs
1	AS1411 aptamer–carbon dots	Spectrofluorometric detection of nucleolin-associated cancer cells	[60]
2	AS1411 aptamer–PEI carbon dots	AS1411-mediated uptake for targeted cancer-cell imaging	[61]
3	Anti-CEA biofunctionalized carbon dots	Electrochemical detection through CEA immunoreaction	[65]
4	Carbon-nanodot ECL biosensor	Electrogenerated-chemiluminescence detection of miRNA-21	[68]
5	Folate-functionalized fluorescent carbon nanoparticles	Imaging of folate receptor-positive cancer cells	[62]
6	Nucleolin-targeted ratiometric carbon dots	Nucleolin-targeted imaging with a cathepsin B readout	[63]
7	Sulforaphane-conjugated carbon dots	Targeted imaging and inhibition of EGFR-overexpressing cells	[64]
8	Aptamer-conjugated carbon nanomaterials	Cancer and bacterial theranostic design evidence summarized in a review	[67]

Any benefits attributed to decorated CNDs are conditional on formulation-specific evidence and cannot be transferred automatically to CBD-loaded CNDs. The examples in Table 5 and the wider CND literature [60,61,62,63,64,65,67,68,69,87,88] show that marker choice depends on the exact target, readout, and application. Researchers must screen ligand density, retained affinity, nonspecific binding, stability, and performance against appropriate controls. This evidence supports the adaptability of decorated CNDs as a research platform, but not a general claim of diagnostic accuracy or therapeutic advantage. Figure 4 illustrates a proposed fluorescence-assisted CND diagnostic workflow; its performance remains unvalidated.

**Figure 4:**
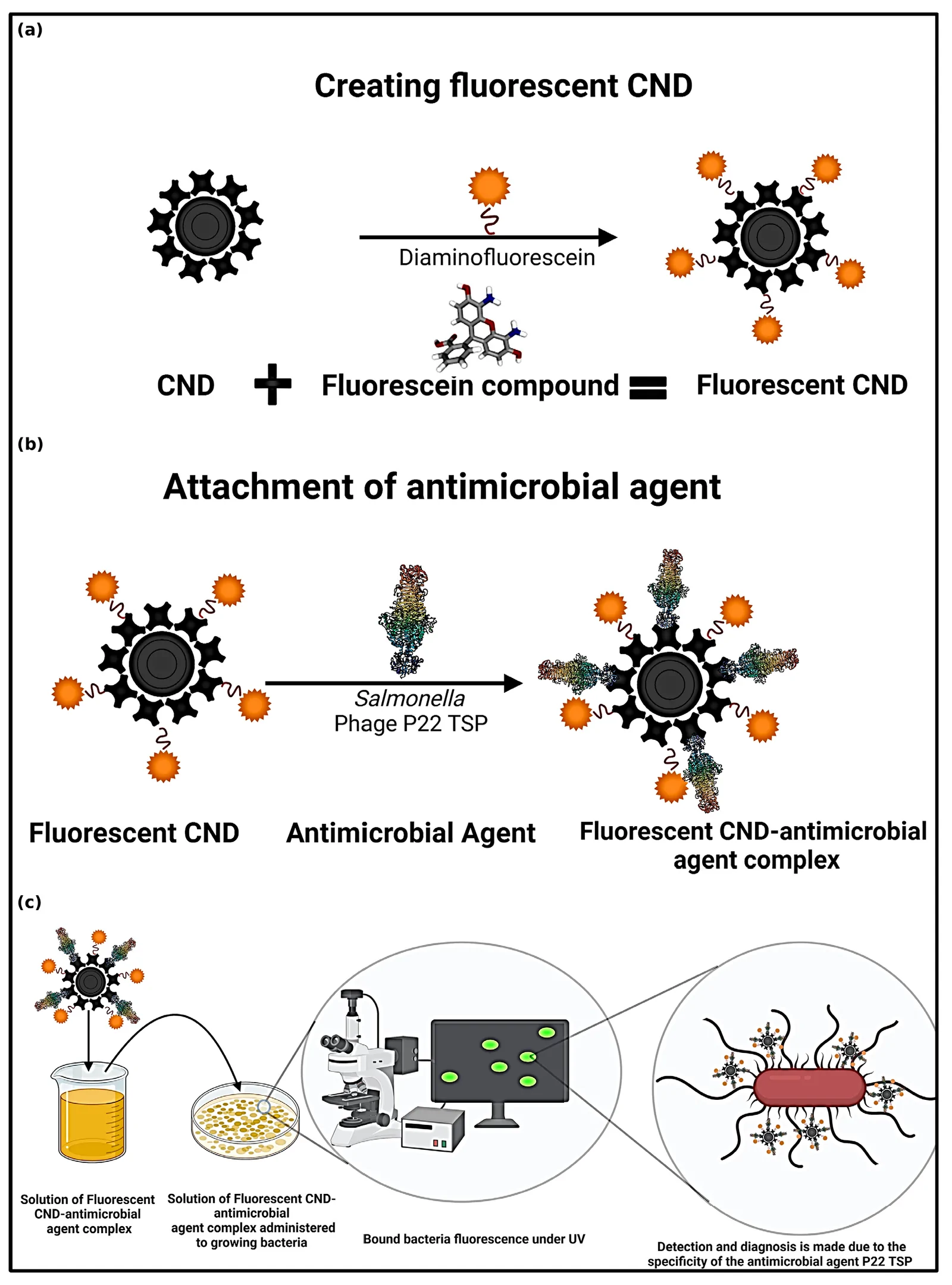
Proposed fluorescence-assisted CND antimicrobial and diagnostic workflow. (**a**) Proposed functionalization of a CND with 4,5-diaminofluorescein (DAF-2; PubChem CID 10666340) to generate a fluorescent construct for tracking in vitro or in vivo. DAF-2 is a nitric oxide-responsive probe [89]; its attachment to CNDs, fluorescence stability, and specificity would require experimental confirmation. (**b**) Proposed complexation of the fluorescent CND with an antimicrobial agent, exemplified by the Salmonella phage P22 tailspike protein (TSP), to track the construct and its interaction with bacteria. P22 TSP carbohydrate binding and its role in host infection have been characterized [90], and orally administered P22 TSP reduced Salmonella colonization in chickens in one study [91]. (**c**) Conceptual detection of Salmonella through P22 TSP interaction with bacterial lipopolysaccharide, followed by fluorescence microscopy visualization. A separate study found antibacterial activity of epsilon 34 phage TSP against CBD-resistant Salmonella strains [92], but that evidence does not validate the proposed P22 TSP–CND diagnostic construct. The scheme remains a testable design and does not establish diagnostic performance or in vivo efficacy.

The marker examples below are platform-design options; receptor expression, binding specificity, uptake, and therapeutic advantage require direct controls.

### 11.4. Diverse Marker Selection

The selection of tissue-specific markers for decorating CNDs is a critical aspect of this proposed approach. Antibodies can bind defined cell-surface antigens; antibody conjugation has been characterized on other nanoparticle platforms [66], and an anti-CEA-functionalized carbon-dot biosensor provides one direct analytical example [65]. Aptamers are single-stranded DNA or RNA molecules selected to bind specific targets through systematic evolution of ligands by exponential enrichment (SELEX) [93]. AS1411 aptamers have been conjugated to carbon dots for cancer-cell detection and imaging [60,61,63], while broader aptamer–carbon-nanomaterial systems are reviewed elsewhere [67]. Short peptides may also recognize defined receptors, as illustrated by RGD–integrin and CD133-binding studies [74,84]. Each ligand–CND combination nevertheless requires its own binding, competition, uptake, and stability controls.

Small organic ligands can also serve as targeting markers when their receptors are experimentally confirmed. Folate–folate receptor targeting is supported in oncology literature and in a fluorescent carbon-nanoparticle imaging study [62,76], while glycyrrhetinic acid-functionalized nanoparticles have been studied for liver cancer delivery [81]. Transferrin receptor targeting is established in broader cancer-delivery literature, including non-CND liposomes [77,85]. Targeting strategies for superparamagnetic iron oxide nanoparticles provide another non-CND comparison and cannot be transferred directly to CNDs [94]. Nanobodies, or single-domain antibodies, are smaller than conventional antibodies and have documented diagnostic applications [95]. These sources support candidate selection and test design; they do not prove targeting or internalization by a CBD-loaded CND.

### 11.5. Enhanced Precision

Tissue-specific markers can improve selectivity in particular experimental systems, but precision cannot be assumed for a decorated CBD–CND formulation. Direct CND studies report target-linked detection, imaging, or uptake for defined constructs [60,61,62,63,64,65,68,69], and broader targeting literature provides receptor and ligand precedents [72,73,74,75,76,77,78,79,80,81,82,83,84,85]. A valid CBD–CND test requires ligand-dependent binding, receptor-positive and receptor-negative controls, competition experiments, matched CBD and CND doses, and quantitative biodistribution. Only a demonstrated increase in target exposure with acceptable off-target exposure would support a targeting claim.

Improved adherence, faster therapeutic response, lower dose, reduced toxicity, and personalized treatment are possible downstream consequences of successful targeting, not properties conferred by marker attachment alone. Evidence for antibodies, aptamers, peptides, folate, transferrin, hyaluronic acid, glycyrrhetinic acid, PSMA ligands, and CD133/EpCAM-directed approaches is target- and formulation-specific [70,71,72,73,74,75,76,77,78,79,80,81,82,83,84,85]. These clinical or translational outcomes should be evaluated only after target selectivity and exposure have been established for the exact CBD–CND formulation.

### 11.6. Reduced Side Effects

One proposed advantage of tissue-specific markers is reduced off-target exposure. For CBD–CNDs, this remains untested: lower systemic exposure, fewer adverse effects, and improved treatment outcomes require comparative biodistribution, efficacy, and safety data. Active-targeting schemes can guide experimental design [96], but passive tumor accumulation through the enhanced permeability and retention effect is variable and may fail clinically [97]. Controlled-release principles are likewise formulation-specific [98]. Figure 5 summarizes the controls needed to evaluate active targeting and the limitations of relying on passive accumulation.

For the targeting approaches discussed here, the delivery outcomes are prospective and have not been established for CBD–CNDs. Tumor binding, intratumoral CBD release, anticancer activity, and systemic clearance are proposed outcomes that require receptor-specific uptake, pharmacokinetic, biodistribution, efficacy, and toxicology studies. Passive accumulation through the enhanced permeability and retention effect is heterogeneous and is often less predictive in humans than in animal models [97]. Reduced side effects could improve adherence and quality of life, but this benefit cannot be attributed to tissue-specific CBD–CND delivery without comparative exposure and safety evidence [96,98].

### 11.7. Targeting Challenges and Optimization

The integration of decorated CNDs with tissue-specific markers presents a promising approach, but it is not without challenges. Marker specificity, affinity, stability, ligand density, and compatibility with the selected CND must be measured. Reviews of CND cancer applications and surface modification emphasize that synthesis and surface chemistry influence optical behavior, biological interaction, and toxicity [87,99]. Even visible absorption varies with electron-accepting and electron-donating CND structures [100], illustrating why CNDs cannot be treated as a uniform material. Biodistribution must be quantified to determine whether a decorated formulation reaches the intended tissue while limiting nonspecific accumulation.

CND size, surface charge, functional groups, purity, and aggregation can influence circulation, cellular uptake, clearance, and toxicity. Ligands must retain binding affinity after conjugation, and the final product must be characterized rather than inferred from its separate components [87,99]. Translation also requires reproducible scale-up, storage stability, quality controls, and formulation-specific safety testing. Available carbon-nanomaterial biosafety literature provides design considerations but cannot substitute for testing the exact decorated CBD–CND product [101]. Multidisciplinary formulation, analytical, toxicology, pharmacology, clinical, and regulatory expertise will be needed before personalized or clinical applications can be considered.

## 12. Comparative Analysis of CBD-Loading Methods

When determining a candidate method for loading CBD onto CNDs, physical association, covalent attachment, and adsorption should be compared using the same CBD batch, CND dose, analytical recovery, and release conditions. Method selection depends on CBD stability, CND surface chemistry, desired route, target exposure, and whether intact CBD must be released. No identified study directly compared these methods for CBD–CNDs; therefore, the discussion below defines experimental decision criteria rather than ranking an established best method.

Noncovalent loading may preserve CBD structure but could allow premature desorption; covalent attachment may improve retention but introduces linker, cleavage, metabolite, and activity questions; adsorption may be simple but can show medium-dependent capacity or burst release. These trade-offs should be evaluated using matched controls rather than assumed from other cargos. The preferred method is the one that provides reproducible composition, adequate CBD recovery, route-appropriate release, acceptable safety, and superior exposure or efficacy relative to free CBD and a benchmark carrier.

### Loading Efficiency and Analytical Metrics

Drug-loading analysis should quantify both encapsulation efficiency and loading capacity. Encapsulation efficiency is the percentage of recovered CBD associated with the carrier relative to the initial CBD amount, whereas loading capacity is the mass of associated CBD relative to the recovered CBD–CND formulation. Free CBD should be separated with a validated method, and CBD recovery should be measured by a stability-indicating assay. No measured CBD–CND encapsulation efficiency or loading capacity was identified in the literature search; values from other cargos cannot fill this gap.

The proposed calculations are encapsulation efficiency (%) = (mass of carrier-associated CBD/initial mass of CBD) × 100; and loading capacity (%) = (mass of carrier-associated CBD/mass of recovered CBD–CND formulation) × 100. Results should include uncertainty, replicate number, total mass balance, and sensitivity to pH, ionic strength, protein-containing media, and storage. Release testing should also account for sink conditions and membrane adsorption [102]. These measurements would determine whether a specific CND preparation actually accommodates CBD; they should not be described as achieved outcomes before experimental data are available.

## 13. Challenges and Potential Solutions

### 13.1. Stability and Long-Term Storage

Long-term stability is a required study outcome, not an established property of CBD-loaded CNDs. CBD is sensitive to light, temperature, oxygen, and formulation matrix [18]. As a non-CND quantitative comparator, the CBD-loaded nanostructured lipid carrier reported by Xie et al. had 87.58% encapsulation efficiency, released more than 50% of CBD within 20 min followed by gradual release, mitigated photodegradation, and was reported as stable for 42 days [43]. A CBD–CND formulation would require its own stability-indicating CBD assay, degradant profile, particle-size and zeta-potential monitoring, loading retention, release comparison, and predefined acceptance criteria under relevant storage conditions.

### 13.2. Safety and Toxicity

Available CND toxicity data show formulation-dependent responses rather than a universal safety profile. In NIH/3T3 fibroblasts, neutral PEG-functionalized carbon dots produced no reported morphology, trafficking, or cell-cycle abnormalities up to 300 µg/mL, whereas pristine negatively charged dots altered reactive oxygen species and cell-cycle behavior and positively charged PEI dots were the most cytotoxic, with effects near 100 µg/mL [103]. A 35-member carbon-dot library tested in human macrophages at 3–200 µg/mL for 24 h showed variable loss of viability, with positive charge, nitrogen content, smaller size, and aggregation associated with greater toxicity [104]. In one animal study, intravenous doses of 5.1 or 51 mg/kg caused no mortality or overt acute toxic signs over 14 days, although this finding applies only to the tested photoluminescent formulation and study design [105]. Conversely, repeated 5 mg/kg subcutaneous administration of compositionally different carbon dots produced formulation-dependent toxicity, including up to 50% lethality for some preparations [106]. None of these CNDs contained CBD. A CBD–CND product therefore requires its own cytotoxicity, genotoxicity, immunotoxicity, hemocompatibility, biodistribution, clearance, and repeated-dose assessment.

### 13.3. Regulatory Considerations

Navigating the regulatory landscape for a novel CBD-loaded CND drug-delivery system would require early definition of product quality, manufacturing controls, nonclinical safety, and clinical evidence. Nanomedicine translation literature emphasizes the importance of reproducible characterization and clinically relevant development pathways [107]. FDA guidance for cannabis and cannabis-derived compounds addresses quality considerations for clinical research, but it does not approve or specifically validate a CBD–CND formulation [108]. Existing studies of CBD activity therefore do not establish regulatory acceptability of a CBD–CND product; regulators would require formulation-specific quality, safety, pharmacokinetic, and efficacy data.

## 14. Current Limitations and Knowledge Gaps

The principal limitation is the absence of direct CBD–CND evidence. No identified study simultaneously established formulation composition, CBD loading, release, stability, pharmacokinetics, biodistribution, safety, and therapeutic performance. Most support concerns either unrelated cargos on CNDs or CBD in chemically different carriers. The discussion above provides a map of possible applications, but those applications should not be interpreted as validated CBD–CND outcomes.

Additional gaps include inconsistent terminology for carbon dots and carbon quantum dots; incomplete reporting of surface chemistry, impurities, and batch reproducibility; uncertain long-term fate; and inadequate comparison with free CBD, unloaded CNDs, physical mixtures, and clinically relevant carrier controls. Targeting and stimulus-responsive release are often proposed but less frequently validated under realistic biological conditions. No clinical evidence was identified for CBD–CNDs.

## 15. Future Perspectives

Future work should begin with a small number of well-characterized formulations selected through predefined criteria. A logical sequence is formulation screening; orthogonal confirmation of loading; release and stability testing; in vitro efficacy and mechanistic controls; dose-ranging pharmacokinetics and biodistribution; short- and long-term safety; disease-model efficacy; and head-to-head comparison with an established CBD delivery platform.

Only after these studies should clinical positioning be considered. Candidate advantages may include combined imaging and delivery, ligand-directed uptake, or route-specific pharmacokinetic improvement. Each proposed advantage must be demonstrated against a suitable comparator, and increased CBD exposure should also be evaluated for altered drug–drug interaction risk [109].

## 16. Conclusions

This review identifies a wide range of possible roles for CNDs in CBD delivery. On critical evaluation, the evidence supports treating these roles as testable hypotheses rather than established therapeutic benefits. CNDs are versatile experimental nanomaterials, and CBD presents a genuine formulation challenge, but no direct preclinical or clinical evidence was identified for a characterized CBD–CND product. The literature therefore does not yet demonstrate CBD–CND synergy, improved bioavailability, targeted delivery, safety, or therapeutic efficacy. Current support is indirect: CNDs have carried other drugs, while non-CND nanocarriers have improved selected CBD formulation and preclinical outcomes. Rigorous characterization, controlled comparative studies, pharmacokinetics, biodistribution, and formulation-specific toxicology are required to determine whether CNDs offer a reproducible advantage for CBD.

## Figures and Tables

**Figure 5:**
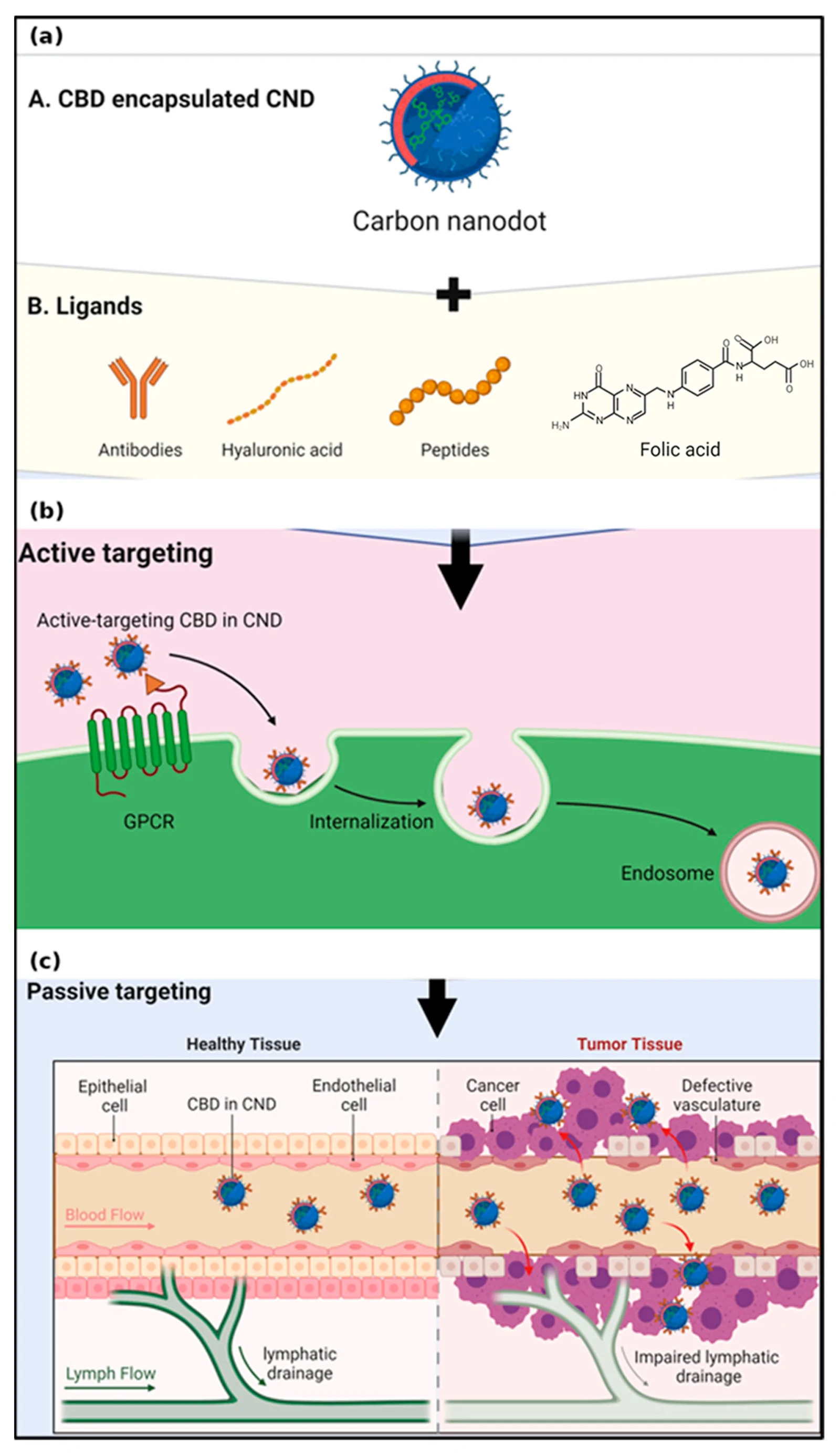
Conceptual targeting routes for CBD-loaded CNDs. (**a**) A CBD-loaded CND may be decorated with candidate ligands such as antibodies, peptides, hyaluronic acid, or folic acid; each ligand must be validated for the intended receptor and final formulation [72,74,76]. (**b**) Proposed active targeting through ligand–receptor binding, cellular uptake, and endosomal internalization. This mechanism requires receptor-positive and receptor-negative controls, competition assays, and direct uptake and release measurements; GPCR activation cannot be assumed for every ligand. (**c**) Proposed passive accumulation through abnormal tumor vasculature and impaired lymphatic drainage. The enhanced permeability and retention effect is heterogeneous and does not ensure tumor delivery, efficacy, or reduced toxicity [97]. These outcomes remain untested for CBD–CNDs.

## Data Availability

No new data were created or analyzed in this study. Data sharing is not applicable to this article.

## Abbreviations

Abbreviation	Definition
BBB	blood–brain barrier
CBC	cannabichromene
CBD	cannabidiol
CBDA	cannabidiolic acid
CBG	cannabigerol
CBGA	cannabigerolic acid
CBN	cannabinol
CND	carbon nanodot
CNS	central nervous system
DAF-2	4,5-diaminofluorescein
DLS	dynamic light scattering
EE	encapsulation efficiency
EGF	epidermal growth factor
EGFR	epidermal growth factor receptor
EPR	enhanced permeability and retention
FRα	folate receptor alpha
FTIR	Fourier-transform infrared spectroscopy
GPCR	G protein-coupled receptor
HER2	human epidermal growth factor receptor 2
HPLC	high-performance liquid chromatography
LC	loading capacity
LC–MS	liquid chromatography–mass spectrometry
MRI	magnetic resonance imaging
PDI	polydispersity index
PK	pharmacokinetics
PLGA	poly(lactic-co-glycolic acid)
PSMA	prostate-specific membrane antigen
TEM	transmission electron microscopy
THC	tetrahydrocannabinol
THCA	tetrahydrocannabinolic acid
TRPV1	transient receptor potential vanilloid 1
